# Revisiting Rosacea Through the Skin–Gut–Brain Axis: A Neuroimmune Perspective

**DOI:** 10.3390/life16020347

**Published:** 2026-02-18

**Authors:** Elvira Lazić Mosler, Marina Vekić Mužević, Dalibor Karlović, Marko Tarle, Marina Raguž

**Affiliations:** 1 School of Medicine, Catholic University of Croatia, 10000 Zagreb, Croatia; elvira.lazic@unicath.hr (E.L.M.); dalibor.karlovic@unicath.hr (D.K.); 2 Department of Dermatology and Venerology, Osijek University Hospital, 31000 Osijek, Croatia; marina.vekic.muzevic@icloud.com; 3 School of Medicine, J.J. Strossmayer University of Osijek, 31000 Osijek, Croatia; 4 Department of Psychiatry, University Hospital Center Sestre Milosrdnice, 10000 Zagreb, Croatia; 5 Department of Maxillofacial Surgery, Dubrava University Hospital, 10000 Zagreb, Croatia; mtarle@sfzg.hr; 6 School of Dental Medicine, University of Zagreb, 10000 Zagreb, Croatia; 7 Department of Neurosurgery, Dubrava University Hospital, 10000 Zagreb, Croatia

**Keywords:** rosacea, skin–gut–brain axis, gut microbiota dysbiosis, neuroimmune interaction, neurogenic inflammation, trigeminal pathways, central sensitization

## Abstract

Rosacea is increasingly recognized as a complex inflammatory disorder extending beyond isolated cutaneous pathology, involving dysregulated interactions between the skin, gastrointestinal system, and central nervous system. The skin–gut–brain axis has emerged as a relevant conceptual framework for understanding this multifactorial disease, integrating gut microbiota dysbiosis, neuroimmune signaling, autonomic nervous system dysfunction, and stress-related mechanisms. The aim of this narrative hypothesis-driven overview is to reframe rosacea as a neuroimmune disorder in which central nervous system involvement plays an active regulatory role, rather than as a purely peripheral or dermatological condition. We synthesize the mechanistically relevant evidence linking gastrointestinal inflammation and microbial imbalance with neurogenic inflammation, mast cell activation, sebaceous gland dysfunction, and aberrant innate immune responses in the skin, with particular emphasis on neurovascular and trigeminal pathways. A key novelty of this perspective lies in highlighting brain-centered mechanisms, including central sensitization, autonomic dysregulation, and stress-related neural modulation, as integral components of the skin–gut–brain axis in rosacea. By integrating peripheral and central processes, we propose rosacea as a model condition for studying neuroimmune dysregulation across interconnected regulatory systems. Finally, we discuss the clinical and translational implications of this framework and outline future research directions, focusing on autonomic regulation, patient stratification, and personalized, multidisciplinary therapeutic approaches.

## 1. Introduction

Rosacea is a chronic inflammatory skin disorder primarily affecting the central face and characterized by persistent erythema, episodic flushing, telangiectasia, inflammatory papules and pustules, and, in a substantial subset of patients, ocular involvement [1,2]. Traditionally, rosacea has also been conceptualized as a disorder of the pilosebaceous unit, with sebaceous glands playing a central role in disease expression through their influence on skin barrier function, lipid composition, and the cutaneous microbiome [2]. Although traditionally classified as a dermatological condition, rosacea exhibits marked clinical heterogeneity and frequently includes prominent neurosensory symptoms such as burning, stinging, dysesthesia, and exaggerated cutaneous sensitivity to physical and chemical stimuli [3,4]. These features, together with the characteristic sensitivity to emotional stress, temperature changes, alcohol, and dietary triggers, strongly suggest that rosacea cannot be fully explained by localized cutaneous inflammation alone.

Over the past decade, the conceptual framework for rosacea has shifted from a rigid subtype-based classification toward a phenotype-driven approach. The Global ROSacea COnsensus (ROSCO) recommendations emphasize the assessment of dominant phenotypic features, such as persistent centrofacial erythema, flushing, inflammatory lesions, phymatous changes, and ocular manifestations, rather than assignment to mutually exclusive subtypes [5,6]. This shift reflects increasing recognition that inflammatory, vascular, and neurosensory components frequently coexist within the same patient and may fluctuate over time. Importantly, a phenotype-based framework provides a clinically intuitive foundation for integrative pathophysiological models that accommodate multisystem involvement.

At the molecular level, rosacea is strongly associated with dysregulation of innate immune responses. Seminal work by Yamasaki and colleagues demonstrated abnormal processing of the antimicrobial peptide cathelicidin, resulting in elevated levels of pro-inflammatory peptide fragments such as LL-37, driven in part by increased activity of epidermal serine proteases including kallikrein 5 [7]. LL-37 has since been shown to promote leukocyte recruitment, angiogenesis, and vasodilation, thereby linking immune activation with vascular alterations characteristic of rosacea [8,9]. These findings firmly established innate immune dysregulation as a core pathogenic mechanism and provided a molecular bridge between inflammation and vascular instability. In parallel, growing evidence supports a central role for neurovascular and neuroimmune mechanisms in rosacea. Clinically, flushing, persistent erythema, and disproportionate sensory discomfort point toward involvement of sensory nerve fibers and neurogenic inflammation [3,10]. Experimental and clinical data indicate that neuropeptides released from cutaneous sensory nerves, including substance P and calcitonin gene-related peptide, can modulate vascular tone, mast cell activation, and immune cell recruitment, thereby amplifying inflammatory responses in the skin [11,12]. This neuroimmune crosstalk provides a biologically plausible explanation for the rapid onset of symptoms following stress or environmental triggers and for the mismatch often observed between subjective symptom severity and visible inflammatory changes. Beyond the skin, gastrointestinal comorbidities have emerged as a recurrent feature in subsets of patients with rosacea. Epidemiological and clinical studies have reported associations between rosacea and functional gastrointestinal disorders, inflammatory bowel disease, and altered gut permeability [13,14]. Of particular relevance, small intestinal bacterial overgrowth (SIBO) has been reported at higher prevalence in patients with rosacea, with interventional studies demonstrating significant improvements in cutaneous symptoms following eradication therapy in breath-test–positive individuals [15,16]. Although these findings do not support a universal gut-driven mechanism, they provide compelling proof-of-concept evidence that gut-derived inflammatory or immune-modulatory signals may contribute to disease activity in specific phenotypes.

The psychosocial and neurobehavioral burden of rosacea further supports the involvement of central regulatory systems. Rosacea is associated with substantial impairment in quality of life and increased prevalence of anxiety, depression, and social avoidance [17,18]. Stress is one of the most frequently reported triggers of flushing and disease exacerbation, implicating central stress-response pathways, including the hypothalamic–pituitary–adrenal (HPA) axis and the autonomic nervous system, in modulation of peripheral inflammation and vascular reactivity [19,20]. This bidirectional interaction suggests a self-reinforcing loop in which visible skin disease, symptom perception, and central stress responses perpetuate one another. Despite extensive investigation into individual pathogenic components, innate immunity, vascular dysfunction, microbial factors, and neurocutaneous signaling, these mechanisms are often discussed in relative isolation. What remains insufficiently explored is an integrative framework capable of connecting gastrointestinal signals, central neural regulation, and cutaneous immune and vascular responses into a coherent systems-level model. The skin–gut–brain axis offers such a framework, integrating gut microbiota and barrier function, neuroimmune communication, and stress-related regulatory pathways [21,22]. Within this context, rosacea may be conceptualized as a neuroimmune disorder characterized by dysregulated communication across peripheral and central systems rather than as an isolated dermatological entity.

While an increasing number of high-quality reviews have explored the gut–skin axis in rosacea, the role of the brain is frequently treated implicitly or confined to stress-related triggers and psychosocial modulation. In contrast, the present review places the central nervous system at the forefront of the proposed skin–gut–brain axis, conceptualizing rosacea as a disorder of dysregulated neuroimmune regulation with active central involvement. By integrating central autonomic control, sensory processing, stress responsivity, and neuroimmune amplification with gastrointestinal and cutaneous mechanisms, this work reframes rosacea as a brain-centered regulatory model of disease expression with peripheral manifestations rather than a primarily gut- or skin-driven condition [12,19,20,21,22,23]. The central hypothesis of this review is that rosacea is not primarily a skin- or gut-limited disorder, but rather a condition of dysregulated neuroimmune regulation, in which brain-driven autonomic, sensory, and stress-related mechanisms integrate and amplify peripheral inflammatory signals in susceptible phenotypes. Within this framework, gastrointestinal dysbiosis and cutaneous immune activation are viewed not as independent drivers, but as interconnected components modulated by central regulatory processes that shape disease expression, symptom perception, and clinical heterogeneity [12,19,20,21,22,23].

Accordingly, the aim of this narrative, hypothesis-driven overview is to revisit rosacea through the lens of the skin–gut–brain axis and to synthesize mechanistically relevant and clinically meaningful evidence linking gut dysbiosis, neuroimmune signaling, autonomic regulation, and cutaneous inflammation. Rather than providing a systematic review, this article seeks to propose an integrative conceptual model with direct implications for phenotyping, multidisciplinary clinical reasoning, and future translational research focused on regulatory networks and patient subgroups most likely to benefit from personalized therapeutic strategies.

## 2. The Skin–Gut–Brain Axis: A Brief Conceptual Framework

The skin–gut–brain axis describes a dynamic, bidirectional communication network linking the gastrointestinal system, the central nervous system, and the skin through neural, immune, endocrine, and microbial pathways. Rather than representing a linear signaling cascade, this axis functions as a complex regulatory system in which peripheral and central inputs continuously modulate immune tone, vascular reactivity, sensory perception, and barrier function [22,23]. Increasing evidence suggests that dysregulation at any node of this network can propagate across systems, resulting in chronic inflammatory or functional disorders with multisystem manifestations.

At the level of the gut, the intestinal microbiota plays a central role in shaping systemic immune responses and neural signaling. Gut microorganisms regulate mucosal barrier integrity, influence antigen exposure, and modulate both innate and adaptive immunity through microbial metabolites, including short-chain fatty acids, bile acid derivatives, and tryptophan metabolites [22,24]. Alterations in microbial composition or increased intestinal permeability may promote low-grade systemic inflammation, providing a source of immune priming that can affect distant organs, including the skin [21,25].

Communication between the gut and the brain is mediated through multiple parallel pathways, including the vagus nerve, spinal afferents, immune mediators, and neuroendocrine signaling via the HPA axis [23,26]. Microbial signals can influence central stress responsivity, emotional behavior, and autonomic balance, while central stress responses, in turn, alter gut motility, secretion, permeability, and microbial composition. This bidirectional regulation is particularly relevant in conditions characterized by stress sensitivity and fluctuating symptom severity.

The skin constitutes both a target and an active participant within this axis. As an immunologically active organ richly innervated by sensory and autonomic nerve fibers, the skin integrates neural signals with local immune responses and vascular regulation [12,27]. Cutaneous immune cells, including keratinocytes, mast cells, and dendritic cells, express receptors for neuropeptides and stress hormones, enabling direct neuroimmune communication. Mast cells, in particular, occupy a strategic position at the interface of nerves, blood vessels, and immune cells and have been implicated as key amplifiers of neurogenic inflammation [12,28].

Neural pathways provide a rapid mechanism for cross-organ communication within the skin–gut–brain axis. Sensory afferent fibers transmit peripheral inflammatory or nociceptive signals to the central nervous system, while efferent autonomic outputs modulate vascular tone, immune cell activity, and barrier function in both the gut and the skin [29,30]. Dysregulation of autonomic balance, particularly increased sympathetic activity or impaired parasympathetic modulation, has been implicated in a range of inflammatory and functional disorders and represents a plausible shared mechanism linking gastrointestinal dysfunction, stress responsiveness, and cutaneous hyperreactivity.

**Figure 1:**
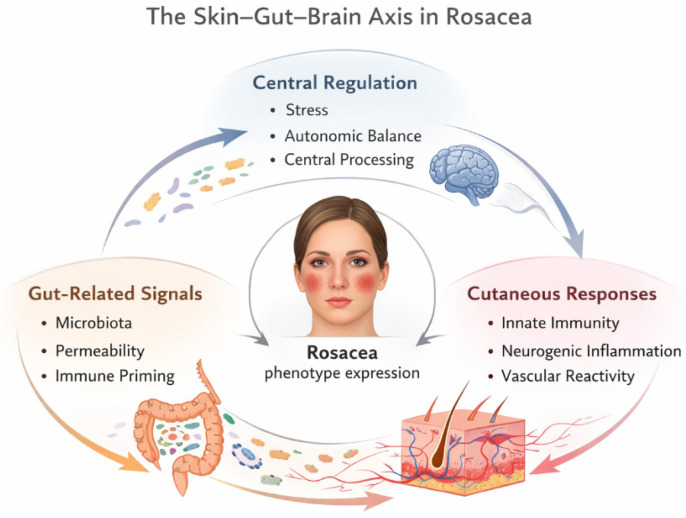
The skin–gut–brain axis in rosacea. This schematic represents rosacea as an emergent neuroimmune condition arising from dysregulated communication within a network linking gut-related immune signaling, central stress and autonomic regulation, and cutaneous inflammatory and vascular responses. Rather than a linear pathway, the skin–gut–brain axis is depicted as a dynamic regulatory system in which bidirectional interactions contribute to disease expression and variability. Figure 1 provides a graphical overview of the skin–gut–brain axis in rosacea and is intended as a conceptual illustration rather than a detailed mechanistic or hierarchical model.

Importantly, the skin–gut–brain axis is increasingly recognized as a framework for understanding diseases characterized by heightened sensory perception and disproportionate symptom burden relative to structural pathology. Conditions such as irritable bowel syndrome, migraine, fibromyalgia, and certain chronic pain syndromes share features of central sensitization, autonomic dysregulation, and immune activation, suggesting overlapping regulatory disturbances rather than isolated organ-specific disease [23,31]. Within this context, inflammatory skin diseases with prominent sensory and vascular components, such as rosacea, may represent peripheral manifestations of broader neuroimmune dysregulation. From a translational perspective, the skin–gut–brain axis offers a unifying model that accommodates heterogeneity, fluctuating disease activity, and variable treatment response. Rather than attributing disease expression to a single pathogenic factor, this framework emphasizes network behavior, feedback loops, and patient-specific vulnerability profiles. Such an approach is particularly relevant for disorders in which conventional organ-centered models fail to fully explain symptom complexity or therapeutic resistance. An integrative view of rosacea as a dysregulated skin–gut–brain network is summarized in Figure 1.

In the following sections, rosacea is examined as a paradigmatic example of skin–gut–brain axis involvement, illustrating how gut-derived immune signals, neurovascular regulation, and central stress-related mechanisms converge at the level of the skin to produce the characteristic clinical features of the disease.

## 3. Rosacea Beyond the Skin: Gut-Related Mechanisms

Growing evidence indicates that rosacea, at least in a subset of patients, is associated with gastrointestinal alterations that extend beyond coincidental comorbidity. Epidemiological studies have reported increased prevalence of functional and inflammatory gastrointestinal disorders among individuals with rosacea, including irritable bowel syndrome, gastroesophageal reflux disease, inflammatory bowel disease, and celiac disease [13,14,32]. These associations suggest that systemic factors originating in the gut may contribute to the inflammatory and neurovascular phenotype observed in rosacea.

One of the most extensively investigated gut-related mechanisms in rosacea is SIBO. In a landmark study, Parodi and colleagues demonstrated a significantly higher prevalence of SIBO in patients with rosacea compared to healthy controls and reported a marked clinical improvement in cutaneous manifestations following eradication therapy in breath-test–positive patients [15]. Importantly, subsequent long-term follow-up confirmed sustained remission in a substantial proportion of responders, supporting a potential causal contribution rather than a transient associative effect [16]. Although SIBO is not universally present in rosacea, these findings provide compelling proof-of-concept that gut microbial imbalance can influence disease activity in a clinically meaningful way. However, it is important to acknowledge that the evidence supporting a causal role of SIBO in rosacea remains limited. Most available studies are observational in nature or involve interventional designs without adequate blinding or control groups, and reported clinical responses to eradication therapy are heterogeneous [15,16]. Moreover, the absence of SIBO in a substantial proportion of patients with rosacea suggests that gut microbial imbalance is unlikely to represent a universal pathogenic driver, but rather a disease-modifying factor relevant to specific clinical phenotypes. Beyond SIBO, alterations in gut microbiota composition and function have been proposed as contributors to rosacea pathophysiology. Emerging microbiome studies suggest that dysbiosis may promote systemic immune activation through increased intestinal permeability, facilitating the translocation of microbial products such as lipopolysaccharides into the circulation [25,33]. These microbial-derived signals can activate pattern recognition receptors, including toll-like receptors, leading to low-grade systemic inflammation that may prime cutaneous innate immune responses. In the context of rosacea, such priming may amplify aberrant cathelicidin processing and downstream inflammatory cascades in predisposed individuals. The gut immune system represents another critical interface linking intestinal dysregulation with cutaneous inflammation. Intestinal inflammation and altered microbiota composition can modulate systemic cytokine profiles, influencing distant immune compartments, including the skin [24]. Experimental models have demonstrated that gut-derived immune signals can shape cutaneous immune tone, supporting the concept of a gut–skin axis mediated by shared immune pathways [21]. In rosacea, this mechanism may help explain why gastrointestinal interventions can lead to improvement of skin symptoms in the absence of direct dermatological treatment.

Beyond generalized dysbiosis, specific gastrointestinal inflammatory conditions have also been examined for their potential contribution to rosacea. Helicobacter pylori infection has been proposed as an additional gut-related factor potentially relevant to rosacea in selected patient subsets. Chronic *H. pylori*-associated gastritis is characterized by persistent low-grade inflammation, altered gastric and intestinal permeability, and systemic immune activation, which may influence inflammatory signaling beyond the gastrointestinal tract [13,14]. Clinical observations suggest that eradication therapy may be associated with improvement of rosacea symptoms in some patients; however, therapeutic response is inconsistent, indicating that *H. pylori* does not represent a universal pathogenic driver. Rather, it may act as an upstream inflammatory stressor capable of modulating disease expression in susceptible individuals through systemic immune priming and autonomic dysregulation [13]. Dietary factors constitute an additional pathway through which intestinal processes may influence rosacea pathophysiology. Alcohol, spicy foods, and high-glycemic diets are known to affect intestinal barrier integrity, gut microbiota composition, and systemic inflammatory tone [21,22,25]. Through these mechanisms, dietary factors may indirectly enhance neurovascular reactivity and neurogenic inflammation, thereby amplifying flushing, erythema, and sensory symptoms characteristic of rosacea. Importantly, dietary influences should not be viewed solely as direct cutaneous triggers, but rather as modulators of gut-immune-neural interactions within the skin–gut–brain axis, consistent with the marked clinical and phenotypic heterogeneity of rosacea [21,22].

Neural pathways further strengthen the gut–skin connection in rosacea. The enteric nervous system and vagal afferents transmit microbial and inflammatory signals from the gut to central autonomic and stress-regulatory circuits [23,30]. Dysregulated gut signaling may therefore influence autonomic balance, favoring sympathetic dominance or impaired parasympathetic modulation, states that are known to exacerbate vascular instability and neurogenic inflammation in the skin. This neurovisceral route provides a mechanistic bridge between gastrointestinal dysfunction, stress sensitivity, and the characteristic flushing and burning sensations reported by rosacea patients.

Importantly, gut-related mechanisms are unlikely to be uniformly relevant across all rosacea phenotypes. Clinical heterogeneity suggests that gastrointestinal contributions may be particularly pronounced in patients with prominent flushing, burning, and stress-responsive disease, whereas purely inflammatory phenotypes may be driven predominantly by local cutaneous factors. This observation aligns with a phenotype-based approach to rosacea, in which gut dysbiosis acts as an upstream modifier of disease expression rather than a universal etiological driver [1,6].

From a translational perspective, the recognition of gut-related mechanisms in rosacea has important clinical implications. Identification of gastrointestinal symptoms, functional bowel disorders, or risk factors for dysbiosis may help stratify patients who could benefit from targeted evaluation and intervention. Moreover, the gut component of the skin–gut–brain axis provides a plausible entry point for multidisciplinary management strategies aimed at modulating systemic inflammatory tone and neuroimmune regulation rather than focusing exclusively on local skin-directed therapies.

## 4. Neuroimmune and Neurovascular Mechanisms in Rosacea

Neuroimmune and neurovascular dysregulation represent central components of rosacea pathophysiology and provide a mechanistic link between cutaneous inflammation, sensory symptoms, and vascular instability. Clinical hallmarks such as episodic flushing, persistent erythema, burning sensations, and heightened reactivity to environmental and emotional stimuli strongly suggest involvement of sensory nerves and neurogenic inflammatory pathways [3,4]. These features are often disproportionate to the degree of visible inflammation, indicating that altered neural processing and neuroimmune communication play a key role in symptom generation.

A pivotal molecular pathway in rosacea involves the dysregulated innate immune response centered on the cathelicidin antimicrobial peptide LL-37. Aberrant processing of cathelicidin, driven by increased activity of serine proteases such as kallikrein 5, results in elevated levels of pro-inflammatory LL-37 fragments that promote leukocyte recruitment, angiogenesis, and vasodilation [7,8]. Importantly, LL-37 has been shown to directly influence vascular endothelial cells and to interact with immune cells, positioning it as a molecular bridge between inflammation and vascular dysfunction in rosacea [9].

Neuroimmune crosstalk further amplifies these processes through the activation of cutaneous sensory nerve fibers. Sensory nerves densely innervate the facial skin and release neuropeptides such as substance P and calcitonin gene-related peptide (CGRP) in response to thermal, chemical, or emotional triggers [12]. These neuropeptides induce vasodilation, increase vascular permeability, and stimulate immune cell activation, contributing to the rapid onset of flushing and edema characteristic of rosacea flares [20,34]. This mechanism exemplifies neurogenic inflammation, in which neural activity directly drives inflammatory and vascular responses in peripheral tissues.

Mast cells occupy a strategic position at the intersection of neural, immune, and vascular networks and have emerged as key amplifiers of neuroimmune signaling in rosacea. Located in close proximity to sensory nerve endings and blood vessels, mast cells respond to neuropeptides and stress mediators by releasing histamine, proteases, cytokines, and vasoactive substances [28]. Experimental models have demonstrated that mast cell activation can potentiate neurogenic inflammation and vascular hyperreactivity, reinforcing the concept of a feed-forward loop between nerves, immune cells, and blood vessels in rosacea-affected skin [12].

In rosacea, persistent cutaneous inflammation profoundly alters sebaceous gland biology. Locally, innate immune activation, neurovascular dysregulation, and microbial-derived stimuli induce sebocyte stress responses, promote pro-inflammatory cytokine release, and generate oxidative damage. These processes result in dysregulated lipid synthesis and qualitative alterations in sebum composition, compromising epidermal barrier function and favoring a pro-inflammatory cutaneous microenvironment. Importantly, altered sebum composition further shapes the skin microbiome, reinforcing inflammatory signaling and sustaining disease activity [8,35].

Beyond local mechanisms, systemic low-grade inflammation associated with gut dysbiosis and stress-related neuroendocrine activation may modulate sebaceous gland function through endocrine and metabolic pathways, including insulin/IGF-1 signaling and androgen sensitivity. In this context, sebaceous gland dysfunction emerges as a downstream effector of dysregulated skin–gut–brain communication, translating systemic neuroimmune imbalance into persistent cutaneous inflammation [12].

The trigeminal sensory system plays a particularly important role in mediating neurovascular responses in rosacea, given the facial distribution of symptoms. Activation of trigeminal afferents can trigger local release of vasoactive neuropeptides and centrally mediated reflexes that influence facial blood flow and immune activation [4]. Functional imaging and clinical observations suggest that altered trigeminal processing may contribute to exaggerated sensory perception and flushing responses, linking peripheral cutaneous changes with central neural regulation [4]. This trigeminovascular involvement provides a mechanistic parallel to other neurovascular conditions characterized by episodic vasodilation and sensory symptoms. Recent neuroanatomical and clinical evidence has highlighted the cervicotrigeminal complex as a key convergence zone integrating trigeminal and upper cervical sensory inputs, with relevance for neurovascular dysregulation and chronic sensory symptoms in disorders of the head and neck [36].

Autonomic nervous system imbalance further modulates neurovascular and neuroimmune interactions in rosacea. Increased sympathetic activity or impaired parasympathetic regulation can enhance vascular instability and promote inflammatory signaling in the skin [19,29]. Stress-induced activation of central autonomic circuits may therefore exacerbate peripheral neurogenic inflammation, contributing to the stress sensitivity and fluctuating course of the disease. This autonomic component integrates central and peripheral mechanisms and reinforces the relevance of the skin–gut–brain axis framework.

**Figure 2:**
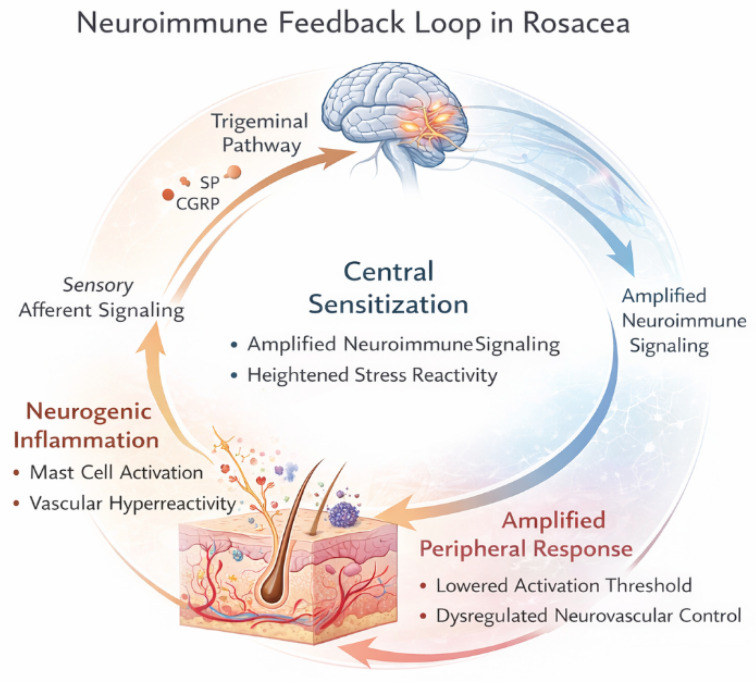
Neuroimmune and neurovascular integration in rosacea. This schematic illustrates a self-reinforcing neuroimmune loop linking peripheral cutaneous inflammation with trigeminal sensory pathways and central processing mechanisms. Activation of sensory nerve endings and mast cells in facial skin promotes neurogenic inflammation and vascular hyperreactivity, while the integration of afferent signals within central trigeminal pathways may facilitate central sensitization and stress-related modulation. Feedback from central regulatory systems can further amplify peripheral responses, contributing to symptom persistence and heightened trigger sensitivity in rosacea.

Collectively, these findings support the view of rosacea as a disorder of dysregulated neuroimmune and neurovascular communication rather than a purely cutaneous inflammatory disease. Aberrant innate immune activation, sensory nerve hyperreactivity, mast cell amplification, and autonomic imbalance converge to produce the characteristic clinical phenotype. Within this framework, neuroimmune dysregulation not only explains the hallmark features of rosacea but also provides a foundation for understanding its association with stress, gastrointestinal factors, and heightened sensory perception. Neuroimmune and neurovascular integration involving trigeminal pathways and central sensitization is schematically illustrated in Figure 2.

## 5. Brain–Skin Interactions: Stress, Autonomic Dysfunction, and Central Sensitization

Within the framework proposed in this review, the brain is conceptualized as an active regulatory hub within the skin–gut–brain axis rather than as a passive recipient of peripheral inflammatory signals. Central neural mechanisms, including autonomic regulation, stress responsivity, and sensory processing, are positioned as key drivers shaping gastrointestinal dysfunction, neuroimmune activation, and cutaneous inflammation in rosacea [12,19,20,21,22,23]. Interactions between the brain and the skin constitute a central component of rosacea pathophysiology and provide a mechanistic explanation for the prominent role of stress, emotional triggers, and disproportionate sensory symptoms observed in many patients. The skin is both a sensory organ and an active neuroimmunological interface, capable of bidirectional communication with central regulatory systems through neural, endocrine, and immune pathways [12,27]. Within this framework, rosacea can be viewed as a disorder in which central stress-related mechanisms modulate peripheral neurovascular and immune responses, contributing to disease onset, persistence, and exacerbation.

Psychological stress is among the most consistently reported triggers of rosacea flares, particularly flushing and burning sensations. Stress activates the HPA axis and the sympathetic branch of the autonomic nervous system, leading to the release of glucocorticoids, catecholamines, and neuropeptides that directly influence immune cell activity, vascular tone, and barrier function in the skin [19,20]. Cutaneous cells, including keratinocytes, mast cells, and endothelial cells, express receptors for stress hormones and neuropeptides, allowing for the rapid translation of central stress signals into local inflammatory and vascular responses [27]. Despite the strong and consistent clinical association between psychological stress and rosacea exacerbation, direct mechanistic evidence linking central stress responses to specific cutaneous inflammatory pathways remains limited. Much of the current understanding is derived from observational studies or extrapolated from related neuroinflammatory and functional disorders, and prospective studies dissecting causal brain–skin interactions in rosacea are still lacking [19,20,27].

Importantly, stress-related neuroendocrine activation also exerts significant effects on gastrointestinal function, providing a critical link between psychological stress, intestinal dysregulation, and cutaneous inflammation. Activation of the hypothalamic–pituitary–adrenal axis and the sympathetic nervous system in response to stress may alter gut motility, increase intestinal permeability, and modulate gut microbiota composition [22,23]. These changes can facilitate translocation of microbial products and promote low-grade systemic immune activation, thereby influencing inflammatory signaling beyond the gastrointestinal tract. Through shared autonomic and immune pathways, stress-induced gut dysfunction may act synergistically with central and peripheral neuroimmune mechanisms to amplify neurogenic inflammation, vascular reactivity, and sensory symptoms in rosacea. This bidirectional interaction positions stress as a central integrative factor within the skin–gut–brain axis rather than as a purely psychological trigger.

Autonomic nervous system dysfunction represents a key mediator of brain–skin communication in rosacea. Altered autonomic balance, characterized by sympathetic predominance and reduced parasympathetic modulation, has been implicated in disorders marked by vascular instability and heightened sensory perception [29,30]. In the context of rosacea, autonomic dysregulation may amplify vasodilation, increase blood flow to facial skin, and facilitate neurogenic inflammation through enhanced release of vasoactive neuropeptides from sensory nerve endings. This mechanism provides a plausible explanation for the rapid onset of flushing in response to emotional or thermal stimuli and for the variability of symptoms across individuals and situations.

Central sensitization further contributes to the clinical phenotype of rosacea, particularly in patients with prominent burning, stinging, or dysesthetic symptoms. Central sensitization refers to increased responsiveness of central nociceptive and sensory pathways following repeated or sustained peripheral input, resulting in amplified symptom perception in the absence of proportional peripheral pathology [37,38]. Convergence of trigeminal and cervical afferent pathways within the cervicotrigeminal complex provides an anatomical substrate for central sensitization and altered sensory processing in craniofacial conditions, linking peripheral neurogenic inflammation with central pain and stress-related circuits [36]. In rosacea, recurrent neurogenic inflammation and sensory nerve activation in facial skin may serve as persistent peripheral input capable of inducing central sensitization, thereby perpetuating symptom severity and lowering the threshold for trigger-induced flares.

The trigeminal sensory system occupies a central position in brain–skin interactions in rosacea. Facial skin is richly innervated by trigeminal afferents, which relay sensory and inflammatory signals to central nuclei involved in pain processing, autonomic regulation, and affective response [4]. Dysregulated trigeminal processing may therefore contribute not only to exaggerated local symptoms but also to altered central integration of sensory input, reinforcing the link between rosacea, stress responsiveness, and emotional state. This trigeminocentric perspective aligns rosacea with other neurovascular and pain-related conditions characterized by episodic vasodilation and heightened sensory awareness.

Clinical observations further support the relevance of central mechanisms in rosacea. Patients frequently report symptom exacerbation during periods of psychological stress, anxiety, or sleep disturbance, and epidemiological studies have demonstrated increased prevalence of anxiety and depressive symptoms among individuals with rosacea [17,18]. These associations are unlikely to reflect mere psychosocial consequences of visible skin disease; rather, they suggest shared regulatory pathways involving stress processing, autonomic control, and immune modulation. Such overlap is consistent with broader models of brain–body interaction in chronic inflammatory and functional disorders.

Within the skin–gut–brain axis framework, brain–skin interactions do not operate in isolation, but are dynamically influenced by gut-derived signals and systemic inflammatory tone. Stress-induced alterations in gut permeability and microbiota composition can further amplify immune activation and autonomic dysregulation, creating a self-reinforcing loop that sustains disease activity [22,23]. This integrative view helps explain why stress management, lifestyle factors, and interventions targeting central regulation may modulate cutaneous symptoms in selected patients, even in the absence of direct dermatological treatment.

Within this framework, central neural regulation is proposed to occupy an upstream position within the skin–gut–brain axis. Brain-mediated autonomic, sensory, and stress-related processes integrate internal and external stimuli and shape downstream gastrointestinal function, immune activation, neurovascular responses, and sebaceous gland activity [12,19,20,21,22,23,29,30]. These peripheral mechanisms are therefore conceptualized not as independent primary drivers, but as context-dependent effectors whose relative contribution to disease expression varies across clinical phenotypes. Such hierarchical organization provides a coherent explanation for the heterogeneity of rosacea manifestations and for the variable dominance of gut-related, vascular, inflammatory, or neurosensory features across patients. Taken together, these findings support a model in which rosacea involves not only peripheral cutaneous pathology but also altered central processing of sensory and stress-related signals. Brain–skin interactions mediated by stress responses, autonomic dysfunction, and central sensitization contribute to symptom perception, trigger sensitivity, and disease chronicity. Recognizing these mechanisms is essential for understanding patient heterogeneity and for developing therapeutic strategies that address regulatory dysfunction rather than focusing exclusively on local inflammatory targets.

## 6. Clinical Implications and Translational Perspectives

Viewing rosacea through the lens of the skin–gut–brain axis has several practical implications for clinical assessment and management. First, this framework supports a more comprehensive patient evaluation that extends beyond cutaneous signs to include gastrointestinal symptoms, stress sensitivity, autonomic features, and sensory complaints. Second, recognition of neuroimmune and neurovascular dysregulation helps explain the frequent mismatch between visible skin changes and symptom burden, thereby validating patient-reported sensory experiences such as burning, flushing, and discomfort.

From a therapeutic perspective, this integrative model suggests that optimal management of rosacea may require individualized, multidisciplinary approaches rather than uniform escalation of topical or anti-inflammatory treatments alone. Addressing upstream modulators, including stress-related autonomic imbalance, gastrointestinal comorbidities, and neurosensory hyperreactivity, may improve symptom control in selected patient subgroups. Importantly, while many of these strategies remain adjunctive and exploratory, the skin–gut–brain axis provides a rational framework for patient stratification and for aligning therapeutic choices with dominant pathophysiological drivers.

Rather than viewing rosacea solely as a localized inflammatory dermatosis, this perspective supports a systems-based approach that acknowledges heterogeneity in disease drivers and symptom expression. Such an approach is particularly relevant for patients with prominent flushing, burning, stress sensitivity, and fluctuating disease activity, in whom conventional skin-directed therapies often yield incomplete or transient benefit.

From a diagnostic standpoint, the recognition of gut- and brain-related contributors encourages broader clinical assessment beyond cutaneous findings. Gastrointestinal symptoms, including bloating, altered bowel habits, reflux, or food-related symptom exacerbation, may signal an underlying gut component relevant to disease modulation. Similarly, heightened stress responsiveness, sleep disturbance, anxiety, or comorbid functional disorders may indicate central regulatory involvement. While routine gastrointestinal or neuropsychiatric screening is not universally warranted, targeted evaluation in selected phenotypes may help identify patients who could benefit from adjunctive or multidisciplinary interventions.

Therapeutically, the skin–gut–brain axis framework supports the concept of layered treatment strategies addressing both peripheral and upstream modulators of disease activity. Conventional topical and systemic dermatological therapies remain essential for controlling cutaneous inflammation and vascular changes. However, in patients with suspected gut involvement, treatment of gastrointestinal dysbiosis or functional disorders has been associated with improvement of cutaneous symptoms in selected cases, underscoring the potential value of gut-directed interventions as adjunctive measures rather than replacements for standard care [13,15].

Central and autonomic regulatory mechanisms represent additional translational targets. Stress-reduction strategies, behavioral interventions, and lifestyle modifications aimed at improving sleep quality and autonomic balance may modulate disease activity by attenuating neurogenic inflammation and vascular hyperreactivity. Although such interventions are often considered supportive rather than therapeutic, their relevance is supported by the strong association between stress, autonomic dysfunction, and symptom exacerbation in rosacea. Importantly, these approaches align with patient-centered care and may enhance treatment adherence and long-term disease control. The neuroimmune perspective also highlights the potential role of mast cell stabilization, modulation of neuropeptide signaling, and targeting of innate immune amplification loops as future therapeutic avenues. While current treatments indirectly influence some of these pathways, emerging research into neuroimmune modulation may open opportunities for more precise interventions aimed at regulatory dysfunction rather than downstream inflammatory endpoints. In this context, rosacea may serve as a clinically accessible model for studying neuroimmune and neurovascular regulation in chronic inflammatory disease.

From a translational research perspective, the skin–gut–brain axis framework supports stratified study designs focusing on patient subgroups defined by dominant phenotypic and systemic features. Rather than treating rosacea as a homogeneous entity, future studies may benefit from integrating gastrointestinal profiles, stress reactivity measures, autonomic markers, and neurosensory symptom burden into clinical phenotyping. Such approaches may improve the interpretation of treatment response variability and facilitate identification of biomarkers reflecting regulatory imbalance rather than static disease severity.

Importantly, this integrative model does not diminish the role of dermatology, but rather expands it, positioning rosacea management at the intersection of dermatology, gastroenterology, and neuroscience. Multidisciplinary collaboration may be particularly valuable for patients with refractory symptoms, complex comorbidities, or pronounced quality-of-life impairment. Within this framework, rosacea exemplifies a condition in which addressing systemic regulation and network behavior may be as critical as targeting local inflammation.

## 7. Future Directions and Hypotheses

Viewing rosacea through the lens of the skin–gut–brain axis opens several important avenues for future research and hypothesis-driven investigation. Rather than focusing exclusively on downstream inflammatory markers or isolated cutaneous features, this framework emphasizes regulatory networks, feedback loops, and patient-specific vulnerability profiles. As such, rosacea may serve as a clinically accessible model for studying neuroimmune dysregulation at the interface of peripheral and central systems.

One key hypothesis arising from this integrative model is that rosacea represents a disorder of impaired regulatory control rather than a primary structural or localized inflammatory disease. In this context, aberrant innate immune activation, neurovascular hyperreactivity, and altered stress responsiveness may reflect a failure of homeostatic mechanisms that normally buffer environmental, microbial, and emotional stimuli. Future studies aimed at quantifying regulatory capacity, such as autonomic balance, stress reactivity, or neuroimmune responsiveness, may therefore provide greater insight into disease susceptibility and progression than static measures of inflammation alone.

Another important research direction involves the identification of biologically meaningful patient subgroups based on dominant axis involvement. Patients with prominent gastrointestinal symptoms, stress sensitivity, or neurosensory complaints may represent distinct phenotypes characterized by upstream dysregulation of gut-derived immune signaling or central neural processing. Integrating gastrointestinal profiling, microbiome analysis, autonomic function testing, and neurosensory assessment into clinical studies may facilitate stratification and improve interpretation of heterogeneous treatment responses. Such an approach aligns with precision medicine paradigms and may help reconcile conflicting findings across rosacea studies.

The skin–gut–brain axis framework also highlights the need for longitudinal and mechanistic studies capable of capturing dynamic interactions over time. Fluctuating disease activity, stress-triggered flares, and variable response to intervention suggest that cross-sectional designs may inadequately reflect the temporal nature of regulatory dysfunction. Longitudinal monitoring of symptoms, stress exposure, autonomic markers, and inflammatory mediators could elucidate causal relationships and feedback loops that sustain disease chronicity.

At the translational level, targeting regulatory pathways rather than isolated inflammatory mediators represents a promising but underexplored strategy. Modulation of autonomic nervous system activity, stress-response circuits, or neuroimmune communication may offer complementary approaches to existing therapies. While such strategies remain largely investigational in rosacea, parallels with other neuroimmune and functional disorders suggest potential benefit in selected patient populations. Importantly, rosacea provides a visible and measurable peripheral readout, making it an attractive model for evaluating interventions aimed at systemic regulation.

Finally, future research should consider the broader implications of skin–gut–brain axis dysregulation for comorbidity and disease overlap. Rosacea shares clinical and mechanistic features with conditions such as functional gastrointestinal disorders, migraine, and chronic pain syndromes, raising the possibility of shared regulatory vulnerabilities. Investigating these overlaps may not only deepen understanding of rosacea but also contribute to a more unified view of neuroimmune disorders affecting multiple organ systems. Together, these hypotheses underscore the potential of moving beyond organ-centered models toward a systems-based understanding of rosacea. By framing rosacea as a disorder of neuroimmune regulation within the skin–gut–brain axis, future research may uncover novel biomarkers, refine patient stratification, and inform personalized therapeutic strategies that address the complexity of this condition.

## 8. Conclusions

Rosacea is increasingly recognized as a complex disorder extending beyond isolated cutaneous inflammation, encompassing dysregulated interactions between the skin, gastrointestinal system, and central nervous system. Evidence from immunological, neurovascular, gastrointestinal, and psychosocial research supports the view that rosacea represents a condition of altered neuroimmune regulation rather than a purely dermatological disease. Within this context, the skin–gut–brain axis provides a unifying conceptual framework capable of integrating heterogeneous clinical features, trigger sensitivity, and disease variability. By synthesizing gut-related immune modulation, neurogenic inflammation, autonomic nervous system imbalance, and central stress-related mechanisms, this perspective highlights regulatory network dysfunction as a core driver of rosacea pathophysiology. Importantly, sebaceous gland dysfunction emerges as a final common pathway through which neuroimmune, gastrointestinal, and stress-related influences converge to shape the clinical expression and heterogeneity of rosacea. Adoption of this integrative framework offers a biologically plausible and clinically meaningful foundation for advancing mechanistic research and refining patient stratification in rosacea.

## Data Availability

No new data were created or analyzed in this study. Data sharing does not apply to this article.

## Abbreviations

The following abbreviations are used in this manuscript:

ROSCO	The Global ROSacea COnsensus
SIBO	small intestinal bacterial overgrowth
HPA	hypothalamic–pituitary–adrenal
